# The core trainee ‘residential’: an opportunity for trainees to feel connected in a world of virtual teaching

**DOI:** 10.1192/bjb.2021.61

**Published:** 2022-12

**Authors:** Angharad de Cates, Victoria Lane, Erin Turner

**Affiliations:** 1 University of Oxford, UK; 2 Birmingham Women's and Children's NHS Foundation Trust, UK; 3 Birmingham and Solihull Mental Health NHS Foundation Trust, UK

**Keywords:** Education and training, trainee support, virtual learning, cognitive neuroscience, residential

## Abstract

MRCPsych courses play a key role in helping trainees prepare for Membership examinations and specialist training. Historically, their social aspect, although arguably as important, has perhaps not been sufficiently prioritised. During a pandemic, when teaching is largely delivered virtually, the social benefit of meeting peers is highlighted by its absence. Given the future likelihood of increased virtual teaching, it is of paramount importance to explore ways of enhancing the sociability of teaching courses. In addition to the social needs of trainees, there is a recognised need to increase and integrate the neuroscience component of the curriculum to better meet the needs of modern day mental health research, treatment and practice. This article describes how the Birmingham MRCPsych course successfully addressed both these issues with a 2-day ‘residential’ in October 2019, and considers whether future residentials could be delivered virtually.

The Birmingham MRCPsych course has a proud tradition of delivering high-quality and innovative teaching from local experts alongside a strong patient and carer contribution.^1^ The course has evolved in response to trainee feedback, with a greater emphasis on Clinical Assessment of Skills and Competencies (CASC) practice and preparing CT3 trainees for higher training. However, reports emerged that some trainees found it difficult to get to know their peers during teaching, because of minimal opportunity for social interaction. The sense of isolation induced by a course that is perceived as ‘unfriendly’ is perhaps most potent for international medical graduates, those returning after prolonged leave and those working in isolated units. Recognising the importance of peer support for psychiatry trainees^2^ the course organiser (E.T.) explored ways to help trainees feel better connected and supported by improving the sociability of the course. The general practitioner (GP) trainees’ positive accounts of their annual residential prompted the course organiser to arrange a meeting with her GP Vocational Training Scheme trainer colleagues to discuss their experiences. Unfortunately, there is limited literature on trainee benefits from a residential; however, the GP trainers universally reported positive outcomes in terms of improved peer support, strengthened trainer–trainee relationships and the ability to cover a topic in more depth. These positive outcomes motivated the Birmingham MRCPsych course to organise their own 2-day residential for core trainees. The proposal received the backing and support of the West Midlands School of Psychiatry Board and funding from Health Education England (HEE).

For over 15 years, there has been global recognition of the need for psychiatrists to have familiarity with the neuroscientific underpinnings of mental illness, necessitating teaching to be delivered to trainees in this area.^3,4^ In 2018, the Royal College of Psychiatrists (RCPsych) Gatsby/Wellcome Neuroscience Project led an update of the core trainee curriculum to emphasise the integration of neuroscience within existing components, specifically focussing on material required for Paper A.^5^ A focus on neuroscience during the residential was considered an initial step to improving the neuroscience content within the MRCPsych programme.

## Aims

The primary aim of the residential was to help trainees feel more connected, supported and integrated with their peers. A secondary aim was to promote neuroscience and its integration into clinical psychiatry, in response to the updated neuroscience component of the 2018 revised syllabus.

To achieve the primary aim, a number of activities promoting team building and belonging were incorporated into the residential, including fun and challenging team games, workshops delivered by higher trainees, a three-course meal, an after-dinner talk from an engaging consultant and a quiz. The ability to work collaboratively and respectively in a team, with both patients and colleagues, is an important part of the General Medical Council's Guidance of Good Medical Practice.^6^ The activities chosen supported the development of interpersonal skills, self-awareness, problem-solving and decision-making within a team environment, supporting trainees to develop valuable skills that have been shown to improve team attitudes, behaviours and clinical accuracy.^7^ For the secondary aim, we drew on the expertise of our West Midlands neuroscience champion (A.d.C.) and the project manager from the RCPsych Gatsby/Wellcome Neuroscience Project (Gareth Cuttle) to design an innovative programme. This included a series of talks from psychiatrists and academics about the latest neuroscientific research. Brain anatomy was taught interactively with a modelling compound, and a CASC scenario helped trainees consider how to integrate neuroscience into the clinical setting (for the two-day residential programme, see Supplementary Appendix 1 available at https://doi.org/10.1192/bjb.2021.61).

The residential took place at a hotel outside Birmingham at the end of October 2019, to avoid trainee examinations and allow adequate time for trainees to settle into their new jobs. Trainees were encouraged to stay overnight to maximise the time they could spend getting to know each other; however, it was recognised that for many trainees this was not possible because of other commitments and the need for accommodation to be personally funded. The regular MRCPsych teaching programme was cancelled for that week, to optimise the likely uptake rate by minimising time spent away from clinical duties.

## Method

### Design

Two methods of feedback collection were implemented.

#### Interactive neuroscience feedback

To assess the impact of the neuroscience content, the interactive presentation software Mentimeter (www.mentimeter.com) was used to collect information on the training grade of participants and to explore their understanding and impressions of neuroscience with four questions (designed using a five-point Likert scale from strongly disagree to strongly agree; see Supplementary Appendix 2 https://www.menti.com/1r623khqiz). The survey was designed by A.d.C., peer-reviewed by E.T., and technologically developed by V.L. It was delivered anonymously both at the beginning and the end of the day, using participants’ smartphones, allowing comparison and evaluation of results following a dedicated day of neuroscience teaching. These results were shared in real time with both participants and organisers, facilitating immediate reflection and discussion on the success of the day.

#### Web-based survey

A web-based survey consisting of 18 questions, created using the Bristol Online Survey tool, was emailed to all participants at the end of the residential. This included an evaluation of each aspect of the course (using a five-point scale) and a series of free-text questions exploring the overall success of the intervention, including the teaching quality, effects on trainee cohesion and perceptions of support (see Supplementary Appendix 3). The survey was designed by E.T., peer-reviewed by the Head of the West Midlands Postgraduate School for Psychiatry and approved by a representative from HEE. Results were collected and collated anonymously by HEE within a 2-week time frame.

The ‘cooler’ feedback survey provided different information from the ‘hot’ Mentimeter, which was purely neuroscience focused. It allowed trainees to comment on each individual session of the day, using free-text responses to add comments. As it was conducted *post hoc* at a later time, it allowed trainees to complete their responses after a period of reflection. It also covered all aspects of the residential, including day 2 activities, ‘soft goals’ (increased cohesion, networking, support and career development) and the facilities at the venue.

### Participants

The web-based survey was completed on a voluntary basis by core trainees in years 1–3. All course participants were invited to complete the short Mentimeter survey, including core trainees, course organisers and tutors. Ethical approval was not required as the data was collected with the primary purpose of evaluating educational opportunities experienced by trainees as part of the programme. Trainees were given the opportunity to email the senior author if they did not wish their free-text responses to be included within the published article.

### Analysis

The analysis was completed by E.T. and A.d.C., in conjunction with V.L. Scaled responses were examined numerically, with free-text responses analysed thematically. The first step of qualitative analysis focused on each researcher becoming familiar with the data, by reading and re-reading the trainees’ free-text responses to allow familiarisation with the content. After data familiarisation, coding occurred by identifying themes from trainee comments, using an iterative process with repeated comparison of emerging issues of interest until no further themes were evident. Each response could be assigned multiple codes and coded sections of text could be of any length. These themes were then discussed and agreed collaboratively by the two researchers (E.T. and A.d.C.). All trainee responses were then organised and labelled until consensus on categories was achieved. Microsoft Excel 2016 for Windows was used for quantitative analysis.

## Results

### Participants

A total of 88 out of 116 regional core trainees attended the residential. In addition, 14 higher trainees and consultants attended and had the opportunity to complete the Mentimeter questions, but not the web-based survey. For the interactive neuroscience feedback (via Mentimeter), of the 102 participants present (88 core trainees and 14 higher trainees/consultants), 59.8% (*n* = 61/102) completed the pre-course feedback and 46.1% (*n* = 47/102) completed the post-course feedback. The response rate for the web-based survey was 74.9% (*n* = 66/88).

### Quantitative results

#### Interactive neuroscience feedback

Following delivery of the Mentimeter questions at the start of day 1, 61 participants completed the scaled questions and 52 provided their grade. At the end of the day, 47 completed the scaled questions and provided their grade. A greater proportion of consultants completed the post-session questions, potentially because of speaker accumulation. Supplementary Fig. 1 provides details on the training grades of respondents for both forms of feedback.

**Fig. 1:**
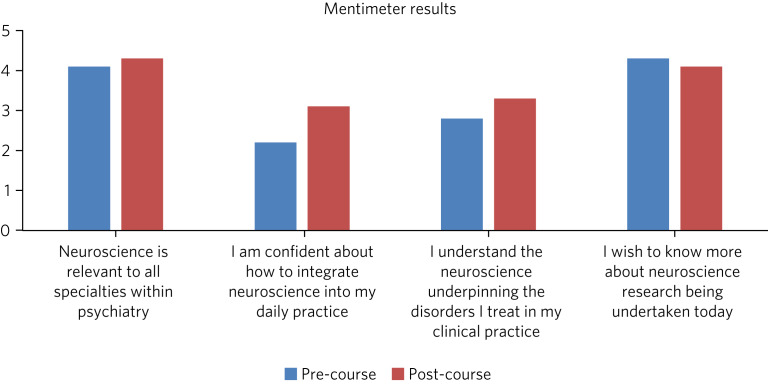
Pre- versus post-residential day 1 Mentimeter responses addressing personal attitudes, using four questions regarding neuroscience in the context of clinical psychiatry.

Figure 1 summarises participant responses to the scaled Mentimeter questions. At the start of the course, most agreed on the relevance of neuroscience to psychiatry (average 4.1/5) and expressed a desire to know more about neuroscientific research (average 4.3/5). However, respondents were less confident when integrating neuroscience into their daily practice (average 2.2/5) and understanding the neuroscience underpinning clinical psychiatric disorders (average 2.8/5). At the end of the course, confidence in these two areas increased to an average of 3.1/5 and 3.3/5, respectively.

#### Web-based survey

A total of 66 trainees submitted the electronic web-based survey completed after the residential. Core trainees contributed the majority of completed survey responses (CT1 *n* = 31, CT2 *n* = 17, CT3 *n* = 13), with further contributions from trainees of a higher grade (MTI *n* = 3, ST4 *n* = 1, ST5 *n* = 1).

Almost all trainees considered the residential to be good or excellent (95.5%, *n* = 63/66), feeling it helped trainees to get to know each other (87.9%, *n* = 58/66) and met the core criteria for the provision of good-quality teaching (87.9%, *n* = 58/66). Further, 77.3% of trainees (*n* = 51/66) felt more supported after the event, and no trainees reported that they would not recommend this course to future trainees.

Almost half of trainees were able to stay at the venue overnight (47%, *n* = 31/66), with a greater proportion (65.2%, *n* = 43/66) asserting that overnight stays were important despite lack of funding for this from HEE. All the trainees who responded cited the value of an overnight stay to be improved team building and social opportunities, and/or minimising long travel times.

**Fig. 2:**
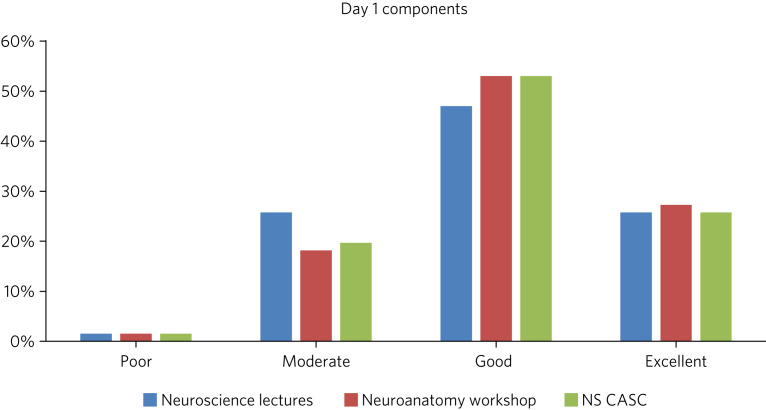
Feedback rating by trainees of individual components of residential day 1 (percentage excellence of rating for each component). NS CASC, Neuroscience Clinical Assessment of Skills and Competencies.

**Fig. 3:**
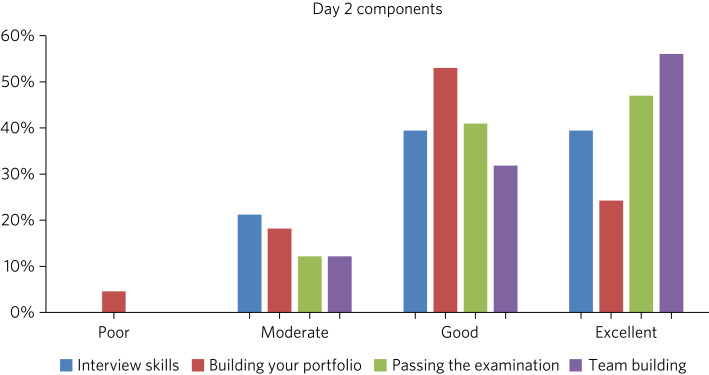
Feedback rating by trainees of individual components of residential day 2 (percentage excellence of rating for each component).

There were three key components to day 1 covered by the feedback survey: the neuroscience lectures, the neuroanatomy practical session and the neuroscience CASC station. The overwhelming majority of trainees found all sessions to be useful (see Fig. 2). The other components covered were interview skills, building your portfolio, helping pass the CASC examination and team building. Three trainees rated the building your portfolio workshop ‘poor’, with no trainees using this grading for any of the other three sessions (see Fig. 3).

There appeared to be some preference of trainees for practical and curriculum-focused sessions, but feedback was not unanimous regarding any single feature. In general, the majority of trainees seemed to find each session ‘good’ or ‘excellent’ for utility and achieving its objectives.

### Qualitative results

Six themes emerged: social benefits, neuroscience content, other content, attitudes, venue and future residential suggestions.

#### Social benefits

The most frequent and almost universally mentioned highlight by trainees was the social aspect of the residential, viewed as an ‘excellent opportunity for trainees to socialise outside of work’ (respondent CT3_58) ‘in a relaxed environment’ (respondent CT3_55). The residential was perceived as ‘very good for team building, making connections, space to unwind with colleagues which is very important. Especially at a time in training which can feel isolated and lonely’ (respondent CT2_27). Socially it was ‘ … . brilliant for CT1s and a great chance to get to know trainees across the Deanery’ (respondent CT2_32). The team-building activity was repeatedly mentioned as a highlight and ‘a great tool to … . get to know colleagues’ (respondent CT1_24).

However, some felt that there was not enough dedicated time to socialising.

‘I felt the event was a bit crammed and tiring at times, which made it harder to engage. Possibly could break up the day with more substantial breaks - was a shame not to use the hotel facilities and may have helped with the team building too! It was suggested also that having the team-building activity on day 1 would have been preferable’ Respondent CT2_48.

#### Neuroscience content

The day devoted to neuroscience was identified as a highlight by many trainees, especially the ‘informative and interesting’ (respondent CT1_53) lectures delivered by ‘high-quality’ (respondent CT1_5) experts alongside ‘thought-provoking neuroscience updates’ (respondent CT3_64). A particular highlight was the brain-making modelling activity, as was the question and answer session at the end of the day: ‘Educationally I felt the Q & A at the end of the day was great’ (respondent CT2_48).

Suggestions for improvement of the neuroscience content were for fewer, shorter lectures with more clinical and examination focus. It was also felt that perhaps some of the lectures were too complex for CT1 trainees:

‘Some lectures were very research orientated, when perhaps more clinical focus would have been helpful…it would be good to have lectures more relevant to exams…spend more time learning what the different parts of the brain are responsible for’ Respondent CT2_52.

#### Other content

The usefulness and relevance of trainee ‘workshops’ on examination preparation, interview skills and e-portfolios were emphasised by several trainees, and it was suggested that a future residential could ‘devote more time to workshops’ (respondent CT3_23). The after-dinner talk by a psychiatrist recounting her personal experience of being a consultant was described as ‘very encouraging and inspiring’ (respondent CT2_8), and the after-dinner quiz was highlighted as ‘fun’ and ‘a good way of working together’ (respondent CT2_51). The residential was viewed as an ‘opportunity to discuss topics that are not routinely focused on in psychiatry training’ (respondent MTI_63).

#### Attitudes

An emerging theme from the trainee feedback was improved attitudes toward psychiatry. Trainees commented that they felt ‘re-enthused about psychiatry’ (respondent CT2_27). Trainees were motivated by ‘lovely, friendly speakers’ (respondent CT1_53) and ‘just seeing people passionate about psychiatry’ (respondent CT1_42).

#### Venue

Trainees appreciated that ‘the entire conference was so well organised. The environment was serene and had calming effect, food was also good. I entirely loved it’ (respondent MTI_33). Trainees appeared to appreciate ‘getting to spend time socialising with other trainees in an informal environment’ (respondent CT3_55).

The overnight stay was not compulsory and those who did not stay felt that perhaps they had missed out: ‘I didn't stay over as the cost for hotel room wasn't sponsored; it would have made the experience richer if I stayed over’ (respondent CT1_46).

#### Future residential suggestions

There was an overwhelmingly positive response to repeating a residential in the future. Topic suggestions for a future residential included ‘trainee well-being and support, psychological therapies’ (respondent CT1_68) and workshops devoted to ‘audit, research and poster presentations’ (respondent CT3_43). It was also suggested that ‘attendance of more senior colleagues and sharing their experiences would be beneficial’ (respondent CT2_66).

### Clinical implications

Our results suggest that a 2-day residential is a highly effective way of improving the sociability of, and sense of belonging to, an MRCPsych course or similar teaching programme. The residential provided an opportunity for trainees to foster peer relationships and establish connections with trainees and trainers across the Deanery, enhancing a sense of ‘belonging’. ‘Belonging’ is prioritised alongside autonomy and competence as one of three core needs of doctors by a recent General Medical Council report,^8^ and may act as a buffer to some of the everyday stresses that trainees experience.

The residential also provided learning opportunities to study a particular topic – in this case neuroscience – in greater detail. Our results showed that trainees’ understanding and confidence grew in this subject.

The positive feedback received following this novel residential course suggests that overall, it was effective at improving trainee cohesion and promoting opportunities for the deeper integration of neuroscience into the Birmingham MRCPsych teaching programme.

### Strengths and limitations

To maximise the number of trainees able to benefit from the full 2-day residential, the planning phase considered and removed a number of obstacles to attendance; a pleasant and easily accessible location was identified, the regular MRCPsych teaching was cancelled for that week and the course was delivered in October, on a date distanced from both trainee examinations and the start of new placements. The lack of funding for overnight accommodation was highlighted in the feedback as a barrier to team building and socialising. Unfortunately, this element was not included in the funding from HEE, as their policy is to no longer fund overnight accommodation for venues <50 miles from home. It would have been helpful to gather information on the reasons that trainees chose not to stay overnight, such as personal finances and child care responsibilities. In particular, it would have been beneficial to establish if international medical graduates were less likely to stay overnight, to determine if mitigating factors to improve access need to be considered in the future.

The design of the programme benefitted from the enthusiasm and expertise of the RCPsych Gatsby/Wellcome Neuroscience Project, including the project manager, in conjunction with senior trainees and consultants involved in the Birmingham MRCPsych course. To better hold the attention of the audience, sessions were delivered by a range of speakers, who each brought their expertise and passion for their subject to the day.

The two methods used to collate feedback had different strengths and limitations. Mentimeter was a rapid, simple and anonymous approach allowing real-time comparison and discussion of data collected both before and after the neuroscience component of the programme. It acted to highlight the perceived relevance of neuroscience as a subject and demonstrated an improvement in understanding and confidence as a result of the residential. The value of the tool was limited, as only five questions were posed, using a questionnaire that had not been previously validated. Of the 102 participants present, only 59.8% completed the pre-course feedback and 46.1% completed the post-course feedback, raising the possibility of response bias.

The web-based feedback survey provided the opportunity for a contrasting type of detailed feedback, collected in the 2 weeks following the residential. Respondents had the opportunity to reflect on their experience and provide detailed free-text responses, which underwent thematic analysis. Overall, the feedback was excellent, but this was again limited by use of an unvalidated questionnaire and a response rate of 74.9% (*n* = 66/88). In the future, to improve the number of responses, trainees might be required to complete the questionnaire before receiving their certificate of attendance.

### Implications and future directions

The benefits of a 2-day residential confer particular importance in a world of virtual teaching, with potentially reduced opportunities for peer support and belonging. Consequently, we considered how these benefits could be replicated virtually, given the unlikelihood of a face-to-face residential in the imminent future. Drawing on the success of the neuroscience teaching day we propose novel teaching methods by inspirational speakers with a focus on short presentations, videos and virtual break-out rooms to enhance trainee participation. Team-building activities, such as a virtual quiz and treasure hunt, can forge bonds and promote friendship and support between trainees. Although the benefits of staying overnight in a hotel are more difficult to replicate virtually, it is possible to organise ‘online’ stimulating and fun social events, as well as an inspiring consultant ‘after-dinner’ speech.

Given the trainee feedback, we suggest that future residentials, be they virtual or face to face, are likely to benefit from more protected time for informal socialisation, improved consultant and training programme director representation, and a greater focus on trainee well-being and support. Other teaching programmes may wish to consider a virtual or face-to-face residential to help foster a sense of belonging and cohesion. It is hoped that the lasting benefits of the residential are enhanced peer relationships, connectivity and a renewed enthusiasm for psychiatry.

## Data Availability

Data is available from the corresponding author, E.T., upon reasonable request.

## About the authors

**Angharad de Cates** is a senior psychiatry trainee and doctoral fellow with the Department of Psychiatry at University of Oxford, UK. **Victoria Lane** is a senior psychiatry trainee at Birmingham Women's and Children's NHS Foundation Trust, UK. **Erin Turner** is a Consultant Psychiatrist with the Early Intervention Psychosis Service, and Head of Psychiatry Teaching Academy at Birmingham and Solihull Mental Health NHS Foundation Trust, UK.

## Supplementary material

For supplementary material accompanying this paper visit https://doi.org/10.1192/bjb.2021.61. click here to view supplementary material
