# Supplementary material for: The core trainee ‘residential’: an opportunity for trainees to feel connected in a world of virtual teaching
Source: BJPsych Bull. 2022 Dec;46(6):336–41. doi: 10.1192/bjb.2021.61 (PMC9813769; doi:10.1192/bjb.2021.61)
Supplement: Supplementary file 1 [file S2056469421000619sup.zip › S2056469421000619sup001.docx]

Figure 1: Pre- vs. post-day 1 mentimeter respondents by grade (% of total responses)
