# Supplementary material for: The core trainee ‘residential’: an opportunity for trainees to feel connected in a world of virtual teaching
Source: BJPsych Bull. 2022 Dec;46(6):336–41. doi: 10.1192/bjb.2021.61 (PMC9813769; doi:10.1192/bjb.2021.61)
Supplement: Supplementary file 1 [file S2056469421000619sup.zip › S2056469421000619sup002.docx]

Appendix 3 **Web-based Feedback Survey**

1. What is your grade of training? CT1/CT2/CT3/ MTI /Other)
2. How would you rate the core trainee residential (v successful to not all successful - 5 point scale)
3. Did it fulfil its objectives of providing quality teaching (v successful to not all successful - 5 point scale)
4. Did it fulfil its objective of helping trainees get to know each other/improving trainee cohesion (v successful to not all successful - 5 point scale)
5. As a result of this event do you feel supported as a trainee (more/less/about the same)
6. Do you think we should run the residential again next year? (yes/no/don’t know)

Please rate

Day 1

- Neuroscience lectures (v successful to not all successful - 5 point scale)
- Neuroanatomy Workshop (v successful to not all successful - 5 point scale)
- Neuroscience CASC activity (v successful to not all successful - 5 point scale)

Day 2

- Interview skills workshop (v successful to not all successful - 5 point scale)
- Building your portfolio (v successful to not all successful - 5 point scale)
- Helping trainees pass the membership exam (v successful to not all successful - 5 point scale)
- Team building activity (v successful to not all successful - 5 point scale)

Was there any particular highlight? (free text)

Is there anything you would change? (free text)

Was the venue suitable? (free text)

Was paying for accommodation a disincentive to attending ? (yes/no/don’t know)

Is there anything else you would like to add to help us plan future events? (free text)
