# Supplementary material for: The core trainee ‘residential’: an opportunity for trainees to feel connected in a world of virtual teaching
Source: BJPsych Bull. 2022 Dec;46(6):336–41. doi: 10.1192/bjb.2021.61 (PMC9813769; doi:10.1192/bjb.2021.61)
Supplement: Supplementary file 1 [file S2056469421000619sup.zip › S2056469421000619sup003.docx]

Appendix 2 **Mentimeter questions**

1. What is your training grade (CT1 / CT2 / CT3 / MTI / ST4 / ST5 / ST6 / Consultant / Other)
2. Neuroscience is relevant to all specialities within psychiatry
3. I am confident about how to integrate neuroscience into my daily practice
4. I understand the neuroscience underpinning the disorders I treat in my clinical practice
5. I wish to know more about neuroscience research being undertaken today
