# Supplementary material for: The core trainee ‘residential’: an opportunity for trainees to feel connected in a world of virtual teaching
Source: BJPsych Bull. 2022 Dec;46(6):336–41. doi: 10.1192/bjb.2021.61 (PMC9813769; doi:10.1192/bjb.2021.61)
Supplement: Supplementary file 1 [file S2056469421000619sup.zip › S2056469421000619sup004.docx]

Appendix 1: **Residential two day programme**

**Programme Day 1**

| 09:00-09:30 | REGISTRATION and REFRESHMENTS |
| --- | --- |
| 09:30-09:40 | **Welcome and Orientation** – Erin Turner, Angharad de Cates, Gareth Cuttle |
| 09:40-09:45 | **Welcome from the President** – Wendy Burn (via video) |
| 09:45-10:00 | **RCPsych Gatsby Wellcome Neuroscience Project** – Gareth Cuttle |
| 10:00-10:30 | **Neuroscience, models of illness, and the clinical encounter in psychiatry: from Jaspers to Kapur** – Matthew Broome |
| 10:30-11:00 | **Cognition, functional imaging and serotonin: what can help us understand and improve depression?** – Angharad de Cates |
| 11:00-11:20 | COFFEE |
| 11:20-11:50 | **What a psychiatrist needs to know about genetics** – Kimberley Kendall |
| 11:50-12:20 | **Three years with the neurologists doing immunology: what can NMDAR-antibody encephalitis teach us about developing the future clinical neuroscientist?** –  Adam Al-Diwani |
| 12:20-13:10 | LUNCH |
| 13:10-13:40 | **Can inflammation explain treatment resistance in psychiatric disorders? The example of treatment-resistant depression** – Riccardo de Giorgi |
| 13:40-14:40 | Group work: **“Neuroanatomy – but not as you know it”** |
| 14:40-15:00 | TEA |
| 15:00-15:30 | **Prediction is very difficult, especially if it's about the future** – Pavan Mallikarjun |
| 15:30-16:30 | Group work: **Communicating applied neuroscience** |
| 16:30-17:00 | Group discussion: **Review of question wall and questions for speakers** |
| 17:00  18:00 | DRINKS and NETWORKING  DINNER & QUIZ (*Worcester Stafford Room)* with after dinner speaker Lisa Brownell |

**Programme Day 2**

Morning Workshops (Consultant and Senior Trainee led)

- Tips to help pass the membership exams
- Building your portfolio to maximise success
- How to Impress at Interview

Afternoon- Team Building ‘Ultimate GPS Challenge’

Awards and plenary

Feedback completion
